# Novel multi epitope-based vaccine against monkeypox virus: vaccinomic approach

**DOI:** 10.1038/s41598-022-20397-z

**Published:** 2022-09-25

**Authors:** Shaza W. Shantier, Mujahed I. Mustafa, Abdelrahman H. Abdelmoneim, Hiba A. Fadl, Sahar G. Elbager, Abdelrafie M. Makhawi

**Affiliations:** 1 Department of Pharmaceutical Chemistry, Faculty of Pharmacy, University of Khartoum, Khartoum, Sudan; 2 Department of Biotechnology, College of Industrial and Applied Sciences, University of Bahri, Khartoum, Sudan; 3 Faculty of Medicine, Alneelain University, Khartoum, Sudan; 4 Department of Haematology, Faculty of Medical Laboratory Sciences, Al-Neelain University, Khartoum, Sudan; 5 Department of Medical Laboratory, Sudanese Medical Research Association, Khartoum, Sudan; 6 Faculty of Medical Laboratory Sciences, University of Medical Sciences and Technology, Khartoum, Sudan

**Keywords:** Biotechnology, Computational biology and bioinformatics

## Abstract

While mankind is still dealing with the COVID-19 pandemic, a case of monkeypox virus (MPXV) has been reported to the WHO on May 7, 2022. Monkeypox is a viral zoonotic disease that has been a public health threat, particularly in Africa. However, it has recently expanded to other parts of the world, so it may soon become a global issue. Thus, the current work was planned and then designed a multi-epitope vaccine against MPXV utilizing the cell surface-binding protein as a target in order to develop a novel and safe vaccine that can evoke the desirable immunological response. The proposed MHC-I, MHC-II, and B-cell epitopes were selected to design multi-epitope vaccine constructs linked with suitable linkers in combination with different adjuvants to enhance the immune responses for the vaccine constructs. The proposed vaccine was composed of 275 amino acids and was shown to be antigenic in Vaxijen server (0.5311) and non-allergenic in AllerTop server. The 3D structure of the designed vaccine was predicted, refined and validated by various in silico tools to assess the stability of the vaccine. Moreover, the solubility of the vaccine construct was found greater than the average solubility provided by protein-Sol server which indicating the solubility of the vaccine construct. Additionally, the most promising epitopes bound to MHC I and MHC II alleles were found having good binding affinities with low energies ranging between − 7.0 and − 8.6 kcal/mol. According to the immunological simulation research, the vaccine was found to elicit a particular immune reaction against the monkeypox virus. Finally, the molecular dynamic study shows that the designed vaccine is stable with minimum RMSF against MHC I allele. We conclude from our research that the cell surface-binding protein is one of the primary proteins involved in MPXV pathogenesis. As a result, our study will aid in the development of appropriate therapeutics and prompt the development of future vaccines against MPXV.

## Introduction

The newly discovered monkeypox virus (MPXV) is a zoonotic orthopox virus that infects people and results in illnesses with similar characteristics to those observed in smallpox. Monkeypox (MPX) cases have been more often reported globally by the World Health Organization (WHO) since May 2022^1,2^. It is brought on by the monkeypox virus, a member of the family *Poxviridae'*s *Orthopoxvirus* genus^3–5^. Considering half of the world's population without immunity to the *Orthopoxvirus*^6^, Poxviruses may have a strong proclivity to emerge outside of their regular ecological range via transmission to a naive community^1^. Thus, great attention has been devoted to monkeypox as a smallpox-like disease and potential bioterrorism agent^6^.

When this virus initially circulated in the Western Hemisphere in the spring of 2003, it attracted a lot of media interest due to the high number of cases that were detected in the US Midwest^7,8^. Yet, there are about 100 confirmed instances of monkeypox as of June 4, 2022, in the United States, the United Kingdom, and many other European countries. Monkeypox represents a variety of clinical manifestations, including flu-like symptoms, fever, malaise, back pain, headache, and a distinctive rash^7^. There are two clades determined to be circulating the world, the Central African clade and the West African clade. The Central African clade had a case fatality rate of 10.6 percent versus 3.6 percent for the West African clade. The United States represents the first country to disclose the two strains of monkeypox with the majority of cases having similar strain to that in the United Kingdom and Europe, with a detected mutation^9^.

MPXV can be diagnosed by real-time polymerase chain reaction (PCR) of samples collected via dry swabs of unroofed lesions or ulcers^10–12^. Seven endemic nations have reported 1408 suspected and 44 confirmed cases between January and June 2022, resulting in 66 fatalities. Cameroon, the Central African Republic, the Democratic Republic of the Congo, Gabon, and Ghana are among the nations where monkeypox is endemic (identified in animals only). The scenario is changing, and WHO predicts that additional cases of monkeypox will be detected as the outbreak advances and surveillance increases in both endemic and non-endemic countries^13^.

Scientists in Portugal have published the first draft genome of the monkeypox virus to an online database on May 19, 2022, as well as other genomes have also followed. These basic genetic research shows that the monkeypox virus strain discovered is connected to a viral strain prevalent primarily in West Africa, when compared to the type that spreads in Central Africa. This strain causes milder symptoms and has a lower death rate of roughly 1% in poor rural populations (which may have a death rate of up to 10%)^14^.

At the molecular level, the monkeypox virus genome consists of a linear double-stranded DNA^15^. The inverted terminal repeats are composed of hairpin loops, tandem repeats, and some open reading frames that are covalently connected at their ends. Despite being a DNA virus, MPXV spends its whole life cycle in the cytoplasm of infected cells. The MPXV genome encodes all of the proteins essential for viral DNA replication, transcription, virion assembly, and egress^16,17^.

There are no vaccines or drugs approved by the USA food and drug administration (FDA) to treat the human monkey poxvirus until 2019. After which, Jynneos was approved for both smallpox and monkey pox. Following that, in November 2021, the Advisory Committee on Immunization Practices (ACIP) recommended the use of JYNNEOS as an alternative to ACAM2000 for primary vaccination against monkey pox, which was in itself has been recommended by the ACIP in 2015 for protection against orthopoxviruses^18^. However, it must be noted that although both vaccines has relative safe profile, they are not synthesized from monkey pox virus itself, which posses a possibility of loss of effectiveness in case of widespread mutations of the virus^19^.

Additionally, Dryvax, a smallpox vaccine, has been used for both smallpox and monkeypox treatment^20^. Nevertheless, the many negative effects influenced both the vaccinated and those in contact with the vaccinated^21,22^.

The capacity of the vaccine to elicit an immune response faster than the virus itself is the fundamental tenet of all immunizations. Even though, traditional vaccines based on biochemical studies have produced potent neutralizing and protective antibodies in the vaccinated animals, they are costly, allergic, and time-consuming, and they require the in vitro cultivation of harmful viruses which raises significant safety concerns^23,24^. On the other hand, the peptide-based vaccine production is exceptionally safe and cost-effective, particularly in comparison to traditional vaccinations. Therefore, this study was dedicated to design a peptide-based vaccine from MPXV cell surface-binding protein using immunoinformatics approach combined with molecular docking studies.

## Material and methods

**Figure 1:**
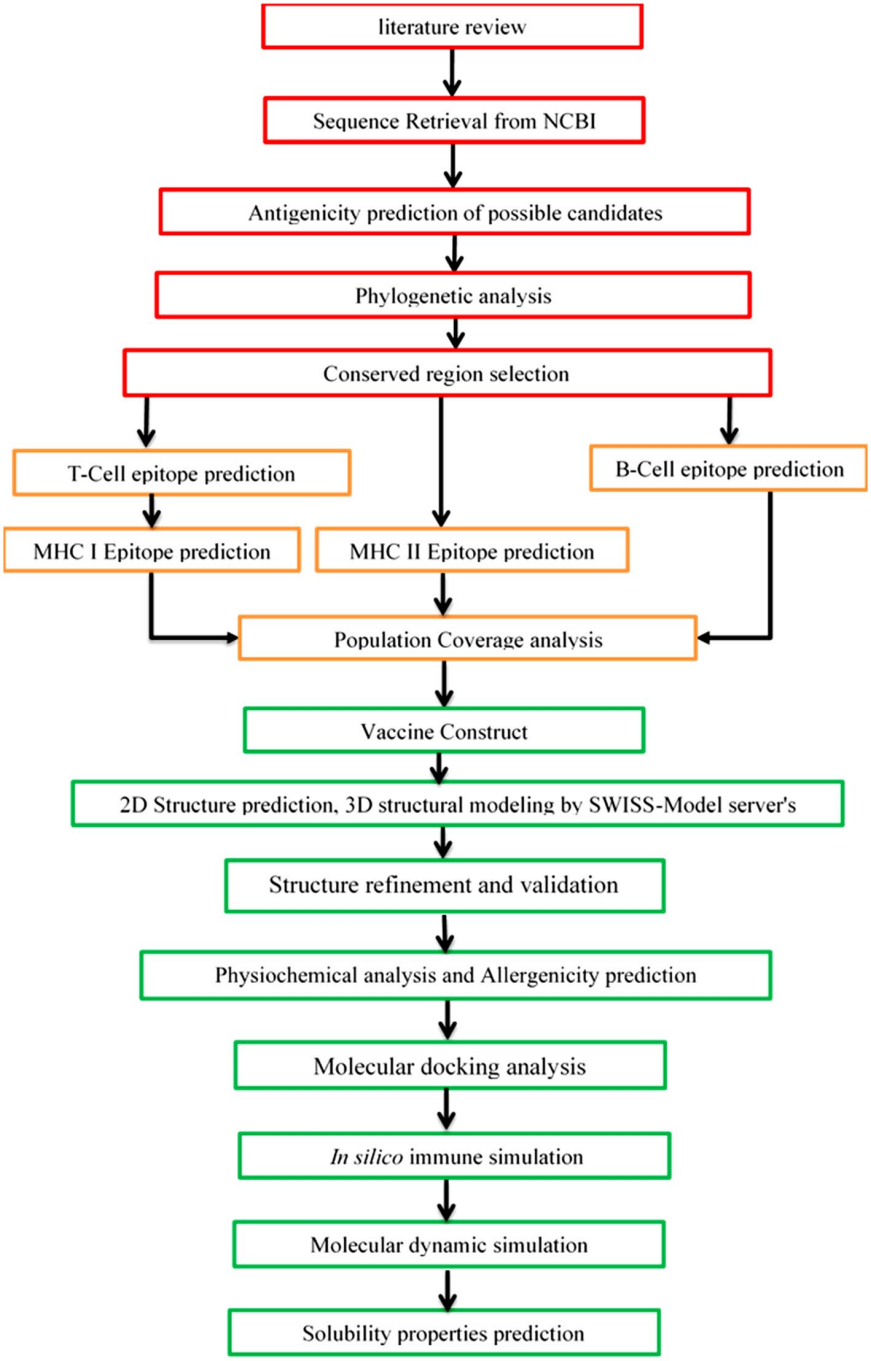
Demonstrated the immunoinformatics approaches used for vaccine design against MPXV.

The Immunoinformatic guided rational design of MPXV Vaccine are shown in Fig. 1.

### Sequence retrieval

The sequence of the cell surface-binding protein of all Monkeypox Virus variants, including those of the new variant strain of USA and Italy were retrieved from the National Center of Biotechnology Information (NCBI) (https://www.ncbi.nlm.nih.gov/) on 17 June 2022.

### Antigenicity prediction

Vaxijen 2.0 (http://www.ddgpharmfac.net/vaxijen/VaxiJen/VaxiJen.html) is a web-based server that predicts the antigenicity using physiochemical properties and ACC methods relying on the alignment independence method. The peptide fragments with a threshold greater than 0.4 were marked as potentially antigenic^25^. This server was thus used to confirm the immunogenic character of all epitopes fragments and the constructed vaccine.

### Phylogenetic analysis

Phylogenetic analysis reveals the evolutionary relationship between the different strains of the Monkeypox virus and trace back patterns of common ancestry between their lineages. Sequence comparison and alignment of the cell surface protein E8L were thus performed using BLASTp (http://blast.ncbi.nim.nih.gov//Blast.cgi?PAGE=Proteins) and Molecular Evolutionary Genetics Analysis (MEGA-X) (version 10.1.18).

### B-cell epitope prediction

In order to create antibodies against disease, B-cells must be activated by particular antigens/epitopes. The BCPRED server (http://ailab.ist.psu.edu/bcpred/) was used to search the indicated antigenic protein of the Monkeypox virus for opportunistic linear B-cell epitopes^26^. The BCPRED service generates 20mer epitopes with a standard specificity of 75% for B-cell receptors using a FASTA sequence as input. For further designing of a multiepitope vaccine, the epitope with the highest score was chosen^26^.

### T-cell epitope prediction tools

The IEDB epitope analysis resource at https://www.iedb.org/home_v3.php was used to predict the MHC class I (http://tools.iedb.org/mhcI), and MHC class II (http://tools.iedb.org/mhcII) binding epitopes. The MHC class I restricted CD8^+^ cytotoxic T lymphocyte (CTL) epitopes from the antigenic viral components of MPXV were predicted using the artificial neural network (ANN) approach^27–29^. All alleles having a binding affinity of IC50 equal to or less than 100 nM were selected for further analysis. Moreover, NetCTL1.2 36^30^, an internet-based server was also used to detect the TAP transport efficiency, proteasomal cleavage, and MHCI molecules binding affinity. The variable was set to 0.5, which has 0.89 and 0.94 sensitivity and specificity, respectively^30^.

In addition, the 15-mer MCH class II-restricted CD4^+^ helper T lymphocyte (HTL) epitopes were predicted using NN-align2.3 as a prediction method. All epitopes that bind to many alleles with IC50 less than 500 nM were selected for further investigations^31^.

### Population coverage

Predicting the distribution of HLA-alleles in the world population is essential for effective multi-epitope vaccine design; thus, the IEDB server (http://tools.iedb.org/tools/population/iedb_input) was used to conduct a population coverage investigation of the selected MHC classes I and II epitopes^32^.

### Multiepitope vaccine construct

To develop an effective molecule capable of inducing an immune response, the production of a vaccine necessitates the presence of three essential components. These components include adjuvants, spacer sequences, and the discovered epitopes (B- and T-cell epitopes). Following internal screening procedures, the anticipated MPXV epitopes were chosen for the final vaccine production. RS09 and PADRE adjuvants were used to improve the stability and activation of both innate and adaptive arms of the immune system. The EAAAK linker was used at the N-terminal site, to link the adjuvants to each other and to the rest of the construct. EAAAK linker improves the stability, and preserve the autonomous function of domains. MHC-I epitopes were linked through AAY which improves the presentation of the epitope. MHC-II epitopes were fused to MHC-I through HEYGAEALERAG linker, and then connected to each other through the GPGPG linker which increases the construct solubility, and flexibility. The KK linker was then used to bind B-cell peptides to help the presentation to antibodies. Finally, the RVRR linker was used to connect to the His-tag sequence at the C-terminal end^33,34^.

### Physiochemical analysis and Allergenicity prediction

ProtParam server available at (https://web.expasy.org/protparam/) was used to predict the physicochemical features of the constructed vaccine and to understand the fundamental nature of the vaccine^35^. The allergenicity of the vaccine was further assessed using AllerTop v 2.0 server^36^.

### Secondary structural prediction

PSIPRED sever (http://bioinf.cs.ucl.ac.uk/psipred/) was used to generate the secondary structures of the constructed vaccine^37^. The PSIPRED is a simple and accurate 2D homology modelling platform that contains two feed-forward neural networks to assess PSI-BLAST output^38,39^.

### Homology modeling and protein validation

The MPXV protein's tertiary structure was created using the SWISS-Model sever. (https://swissmodel.expasy.org/). The obtained model was visualized using Discovery Studio 2020 (https://www.3ds.com/products-services/biovia/products/molecular-modeling-simulation/biovia-discovery-studio/visualization/). The proposed three-dimensional structure was validated using QMEAN. (https://swissmodel.expasy.org/qmean/).

### 3D model refinement and validations

The SWISS-Model server's best protein model was redesigned and refined using the GalaxyRefine web server (http://galaxy.seoklab.org/cgi-bin/submit.cgi?type=REFINE). The molecular dynamic simulation replaces amino acids with high-probability rotamers, resulting in total structural relaxation^40^. The server recommends five refined models that differ in GDT-HA, RMSD, MolProbity, Clash Score, Poor rotamers, and Rama favored. Following that, the improved model required validations on two servers (ProSA-web and PROCHECK servers). The ProSA-web server calculates the total quality score based on a Z-score for all known protein structures; while the protein quality was assessed using the PROCHECK server and the Ramachandran plot (https://www.ebi.ac.uk/thornton-srv/software/PROCHECK/)^41^.

### Immune simulation

C-ImmSim, an online simulation server (https://kraken.iac.rm.cnr.it/C-IMMSIM/) was used to study the generation of adaptive immunity and also the immune interactions. Human host leukocyte antigens (HLA-A*01:01 & A*02:01, HLA-B*07:02 & B*39:01, and HLA-DRB1*01:01 & DRB1*04:01) were selected, and the simulation parameters were left at their default settings for times of 1 h, 84 h, and 168 h. The injection of the vaccine did not contain LPS, and the simulation's volume was set to 10 with a 12,345 random seed. 1000 simulation steps of an immune simulation were run.

### Molecular docking

To ensure the durability of the immune response, developed vaccines must interact with the immune cells. The molecular docking was thus performed utilizing The Major Histocompatibility Complex (MHCs) receptors. For proper computational calculations, the promising epitopes and the target protein were prepared. LigPrep tool interfaced with Maestro module of Schrödinger suite was used for the ligand preparation. The 3 D structures including all possible tautomers and ionization states at pH 7.0 ± 2.0 of all the epitopes were generated and minimized using optimized potential liquid simulations (OPLS4) force field. Schrödinger’s multi-step Protein Preparation Wizard PrepWizard was used for the protein preparations^42^. The high-resolution protein crystal structures of MHC I alleles (HLA-A*02:01, HLA-B*15:01), and MHC class II allele (HLA-DRB1) with PDB IDs 4UQ3, 6VB3 and 6HBY, respectively at 2 Å resolution was retrieved from RCSB Protein Data Bank. Charges and bond orders were assigned, hydrogens were added to the heavy atoms, all water molecules and heteroatoms were then removed. OPLS4 force field was used for optimization and energy minimization for both the epitopes and protein final structures. Glide XP (extra precision) module of Schrödinger Suite was used to dock the promising epitopes into the active site of the crystal structures^42^. Best-scoring docked pose of the molecule obtained was superimposed against X-ray crystal orientation and conformation of the bound ligand and RMSD was calculated. Visualization was then conducted using Schrödinger Suite and DS visualizer client.

### Molecular dynamic simulation

Following molecular docking, MD simulations were performed on two systems: uncomplexed MHC I allele and MHC I allele complexed with the proposed peptide. Molecular Dynamics simulation was utilized to investigate the binding stability of the final complexes using WEBGRO for macromolecular simulations (https://simlab.uams.edu/). Using GROMOS96 43a1 force field parameters, the whole system was solvated in water, neutralized, and 0.15 M salt of NaCl was added. The sharpest descending strategy resulted in an energy decrease of 5000 steps. The kinds of equilibration employed were constant quantity, volume, temperature (NVT/NPT), and pressure. The temperature was set to 300 K and the pressure was set to 1.0 bar for a 100 ns simulation time and 1000 frames each simulation. Root Mean Square Deviation (RMSD) and Root Mean Square Fluctuation (RMSF) were the simulation parameters required. The requested simulation parameters were: Root Mean Square Deviation (RMSD), Root Mean Square Fluctuation (RMSF), Radius of Gyration (Rg), intermolecular H-bonding (H-bonds) and Solvent Accessible Surface Area (SASA). Topology file of the protein–ligand complexes were created using PRODRG sever^43^.

### Solubility properties prediction

Protein-sol (https://protein-sol.manchester.ac.uk/) is a web-based tool for performing numerical simulations and predicting protein solubility^44^. The server was used to estimate the multi-epitope protein solubility when expressed in *E. coli*.

## Results

### Antigenicity prediction and conservation analysis

The alignment of the seven sequences yielded 6 conserved peptides with a length greater than 18 amino acids. The antigenicity was then tested using the VaxiJen server with score threshold for viruses is 0.4, which means that proteins with a score greater than 0.4 are deemed antigenic, whereas proteins with a score less than 0.4 are considered non-antigenic. The VaxiJen score for Monkeypox Virus's tested protein was more than 0.4 (0.5311). Moreover, analysis of those conserved regions showed only four antigenic regions that met VaxiJen's default criterion of 0.4. (Table 1).

**Table 1:** Conserved regions of Cell surface binding protein.

Conserved peptide	Score
MPQQLSPINIETKKAISD	0.9972
RLKTLDIHYNESKPTTIQNTGKLVRINFKGGYISGGFLPNEYVLSTIHIYWGKEDDYGSNHLIDVYKYSGEINLVHWNKKKYSSYEE	0.7807
KKHDDGIIIIAIFLQVSDHKNVYFQKIVNQLDSIRSANMSAPFDSVFYLDNLLPSTLDYFTYLGTTINHSADA	0.4468
WIIFPTPINIHSDQLSKFRTLLSSSNHEGKP	0.0067
YITENYRNPYKLNDDTQVYYSGEIIRAATTSPVRENYFMKWLSDLR	0.1645
CFSYYQKYIEGNKTFAIIAIVFVFILT	0.6078

### Phylogenetic analysis

**Figure 2:**
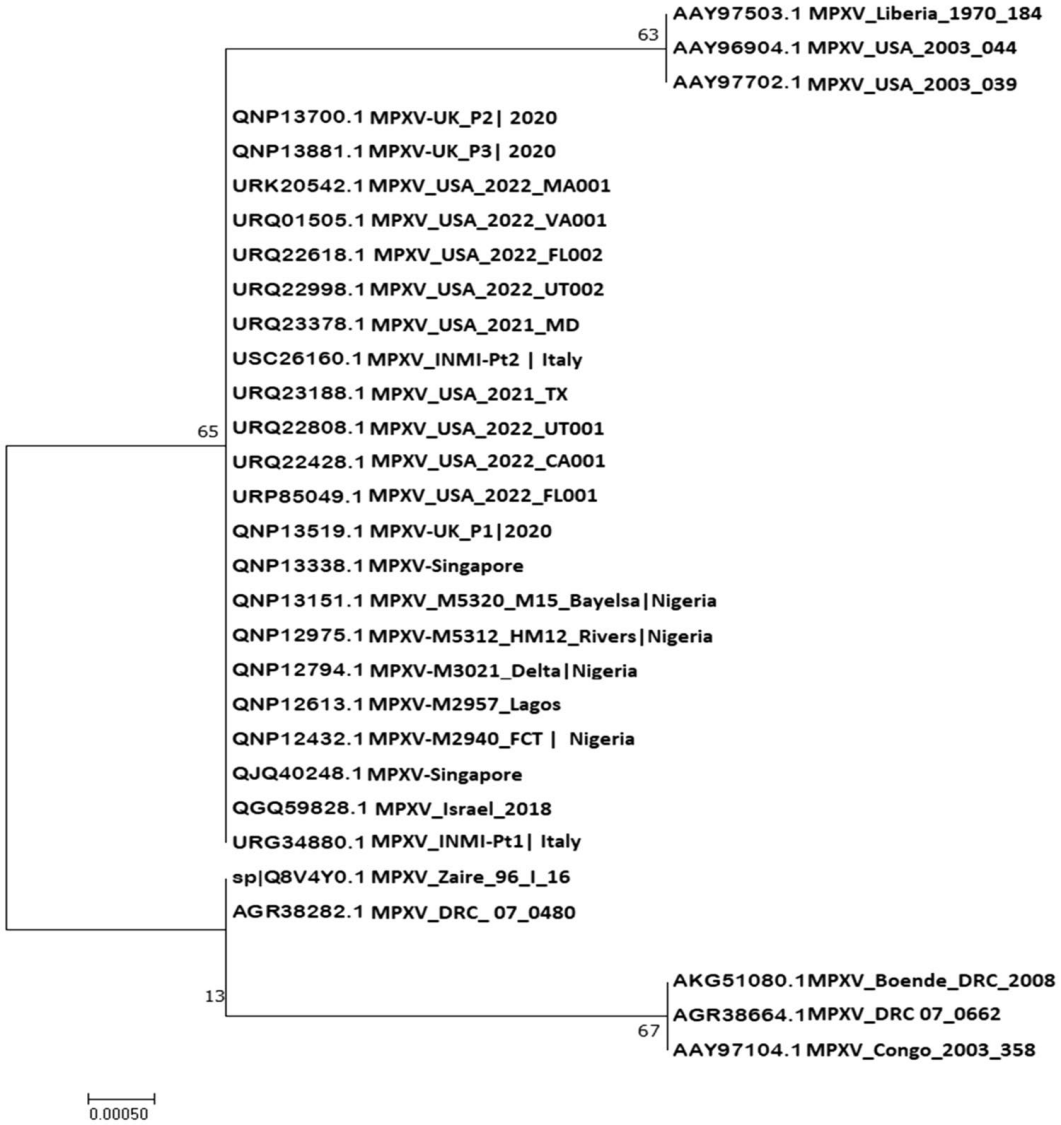
Phylogenetic analysis of the top similar sequences of MPXV.

Sequence comparison and alignment results revealed that E8L protein was conserved, with 98.36% to 100% identity among all Monkeypox virus genomes isolates in till June 8, 2022 (Fig. 2).

### Identification and prediction of B cell epitopes

The BCPRED of B cell proposed 5 epitopes, and all epitopes were found 100% conserved using IEDB conservation tools, and thus can generate immune response (Table 2).

**Table 2:** List of linear B-cell epitopes with their location and score by using the BCPRED server:

Position	Epitope	Score
94	VHWNKKKYSSYEEAKKHDDG	0.966
64	STIHIYWGKEDDYGSNHLID	0.935
238	IRAATTSPVRENYFMKWLSD	0.912
204	SSSNHEGKPHYITENYRNPY	0.891
43	VRINFKGGYISGGFLPNEYV	0.851

### Prediction of T cell epitopes

Tables 3 summarizes the most promising peptides bound to MHC I along with their immunogenicity and allergenicity predication.

**Table 3:** MHC I associated peptides and alleles.

Residue position	Peptide	Alleles	IC50	Allergen	Predicted binding affinity	C-terminal cleavage efficiency	TAP transport efficiency	Combined score
274–282	KTFAIIAIV	HLA-A*02:01, HLA-A*02:06, HLA-A*30:01, HLA-A*32:01, HLA-A*68:02, HLA-C*12:03	57.58	Non-allergen	0.0849	0.5324	0.6530	0.4730
142–150	RSANMSAPF	HLA-A*32:01, HLA-B*15:01, HLA-B*58:01, HLA-C*03:03	5.13	Non-allergen	0.1234	0.3332	2.8450	0.7160
276–284	FAIIAIVFV	HLA-A*02:06, HLA-A*68:02, HLA-C*03:03, HLA-C*12:03	14.07	Non-allergen	0.0772	0.1201	0.3510	0.3633
146–154	MSAPFDSVF	HLA-B*15:01, HLA-B*35:01, HLA-B*57:01, HLA-C*12:03	10.1	Non-allergen	0.2462	0.9564	2.7800	1.3277

Regarding MHC II related peptides. 3256 peptides were retrieved from IEDB server along with the associated alleles. Then each peptide were linked to the alleles associated with and the data was sorted according to the number of associated alleles. Six peptides were found to have the highest number of associated alleles while in the same time having IC-50 less than 500 (Table 4).

**Table 4:** MHC II associated peptides and alleles.

IC50	Start	End	Core Sequence	Combined alleles	Allergen	Alleles NO	Combined score
8.7	234	248	IRAATTSPV	HLA-DQA1*01:02/DQB1*05:01, HLA-DQA1*01:02/DQB1*06:02, HLA-DQA1*02:01/DQB1*03:01, HLA-DQA1*05:01/DQB1*03:03, HLA-DQA1*05:01/DQB1*03:01, HLA-DRB1*04:01, HLA-DRB1*04:05, HLA-DRB1*01:01, HLA-DRB1*13:02, HLA-DRB1*10:01, HLA-DRB1*09:01,HLA-DRB1*08:02, HLA-DRB1*07:01, HLA-DRB3*03:01, HLA-DRB4*01:03, HLA-DRB1*15:01, HLA-DRB3*02:02,HLA-DRB1*16:02, HLA-DRB5*01:01	Allergen	19	0.3878
135.7	248	262	FMKWLSDLR	HLA-DPA1*03:01/DPB1*04:02, HLA-DPA1*02:01/DPB1*01:01, HLA-DQA1*01:02/DQB1*05:01, HLA-DQA1*01:02/DQB1*05:02, HLA-DRB1*04:05, HLA-DRB1*04:04, HLA-DRB1*04:01, HLA-DRB1*13:01, HLA-DRB1*10:01, HLA-DRB1*09:01, HLA-DRB1*16:02, HLA-DRB4*01:03, HLA-DRB4*01:01, HLA-DRB1*15:01, HLA-DRB1*16:02, HLA-DRB5*01:01	Non-allergen	16	0.3910
257.8	195	209	FRTLLSSSN	HLA-DPA1*01:03/DPB1*03:01, HLA-DQA1*01:02/DQB1*05:01, HLA-DQA1*05:01/DQB1*03:03, HLA-DRB1*01:01, HLA-DRB1*04:05, HLA-DRB1*04:01, HLA-DRB1*10:01, HLA-DRB1*08:02, HLA-DRB1*11:01, HLA-DRB1*09:01, HLA-DRB1*07:01, HLA-DRB1*12:01, HLA-DRB1*16:02, HLA-DRB5*01:01, HLA-DRB1*15:01, HLA-DRB3*02:02	Non-allergen	16	0.2164
177.1	59	73	YVLSTIHIY	HLA-DPA1*01:03/DPB1*06:01,HLA-DPA1*01:03/DPB1*02:01, HLA-DPA1*01:03/DPB1*04:01, HLA-DRB1*01:01, HLA-DRB1*04:05, HLA-DRB1*04:01, HLA-DRB1*13:01, HLA-DRB1*07:01, HLA-DRB1*09:01, HLA-DRB1*10:01, HLA-DRB1*08:01, HLA-DRB1*13:02, HLA-DRB3*02:02, HLA-DRB4*01:03, HLA-DRB3*03:01, HLA-DRB5*01:01	Non-allergen	16	1.339
26.1	287	301	FLMSQRYSR	HLA-DPA1*01:03/DPB1*06:01,HLA-DQA1*02:01/DQB1*04:02, HLA-DQA1*06:01/DQB1*04:02,HLA-DQA1*05:01/DQB1*04:02, HLA-DRB1*03:01, HLA-DRB1*01:01, HLA-DRB1*11:01, HLA-DRB1*08:01, HLA-DRB1*10:01, HLA-DRB1*08:02, HLA-DRB4*01:03, HLA-DRB5*01:01, HLA-DRB4*01:01, HLA-DRB1*16:02, HLA-DRB3*02:02	Non- allergen	15	0.4460
29.1	165	179	FTYLGTTIN	HLA-DQA1*01:02/DQB1*05:01,HLA-DQA1*02:01/DQB1*04:02, HLA-DQA1*02:01/DQB1*03:01,HLA-DQA1*02:01/DQB1*03:03, HLA-DQA1*05:01/DQB1*04:02,HLA-DQA1*06:01/DQB1*04:02, HLA-DQA1*05:01/DQB1*03:02,HLA-DRB1*01:01, HLA-DRB1*04:05, HLA-DRB1*04:01, HLA-DRB1*10:01, HLA-DRB1*07:01, HLA-DRB1*08:01, HLA-DRB1*09:01, HLA-DRB5*01:01	Allergen	15	0.2527

### Population coverage

**Figure 3:**
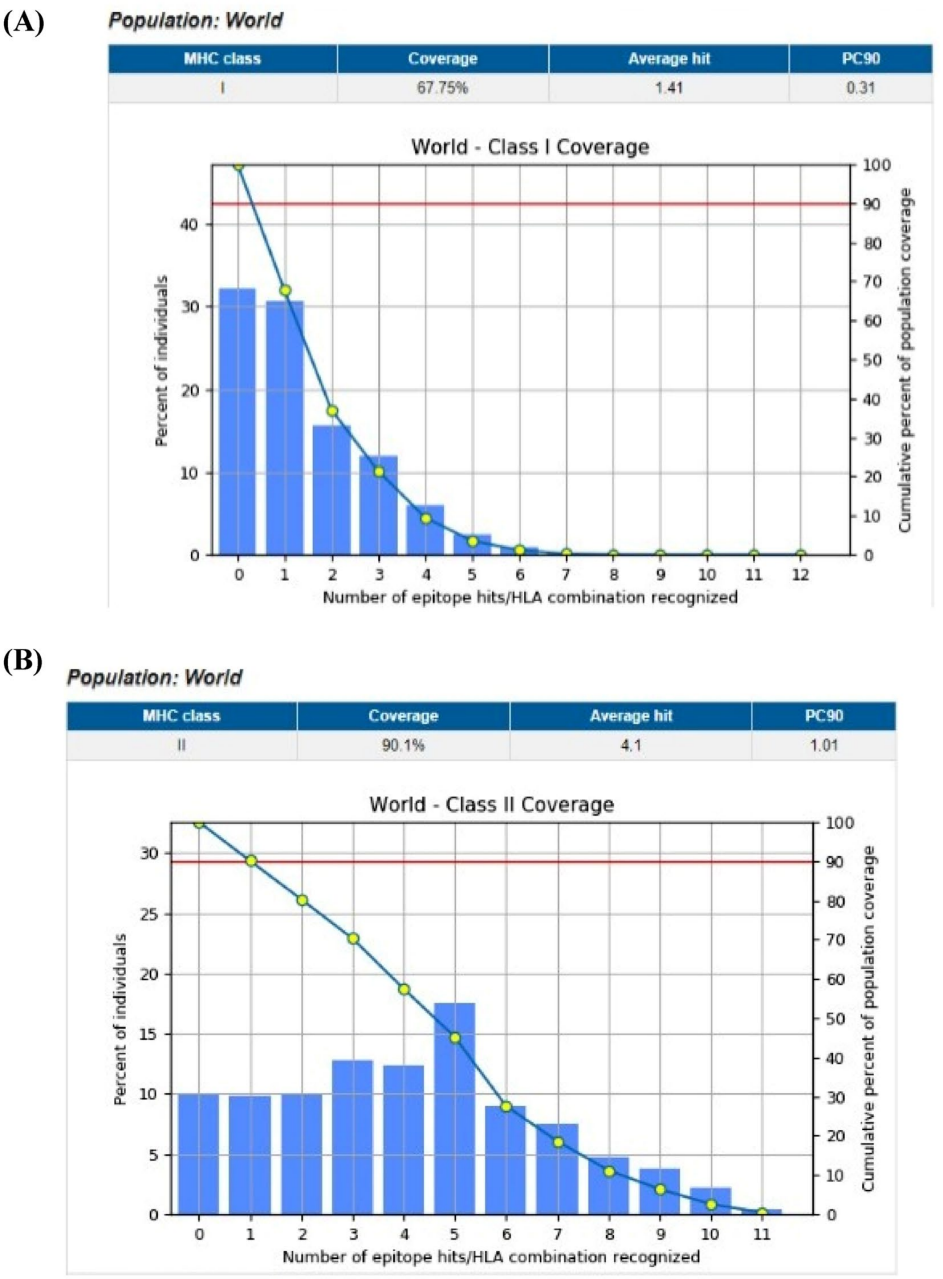
(**A**) Shows the global coverage for the top 10 MHC-I peptides, (**B**) Shows the global coverage for the top 10 MHC-II peptides.

The world coverage for the predicted epitopes are summarized in Fig. 3.

### Vaccine construct

**Figure 4:**
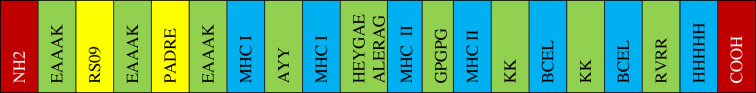
Graphical representation of the Final vaccine construct. (Green: Linkers, Yellow: adjuvant, Dark red: N and C terminal sites, Blue: The chosen peptides for the vaccine).

The most promising epitopes were combined to generate the vaccine construct with the use of the KK, AAY, and GPGPG linkers, respectively. The final 275 peptide vaccine construct is APPHALSEAAAKAKFVAAWTLKAAAKTFAIIAIVAYYRSANMSAPFAYYFAIIAIVFVAYYMSAPFDSVFHEYGAEALERAGIRAATTSPVGPGPGFMKWLSDLRFRTLLSSSNGPGPGYVLSTIHIYGPGPGFLMSQRYSRGPGPGFTYLGTTINKKVHWNKKKYSSYEEAKKHDDGKKSTIHIYWGKEDDYGSNHLIDKKIRAATTSPVRENYFMKWLSDKKSSSNHEGKPHYITENYRNPYKKVRINFKGGYISGGFLPNEYVRVRRHHHHH (Fig. 4).

### Secondary structure prediction

**Figure 5:**
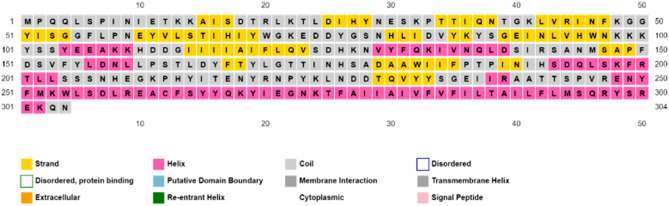
Secondary structure analysis.

PSIPRED was used to predict the secondary structure of the Monkeypox virus vaccine. The alpha helix residues are in pink, the beta strand residues are in yellow and the coil residues are in grey. According to the predicted secondary structure, the final vaccine contains 27.63% alpha helix, 21.71% beta strand, and 42.43% coil (Fig. 5).

### Homology modeling and protein validation

**Figure 6:**
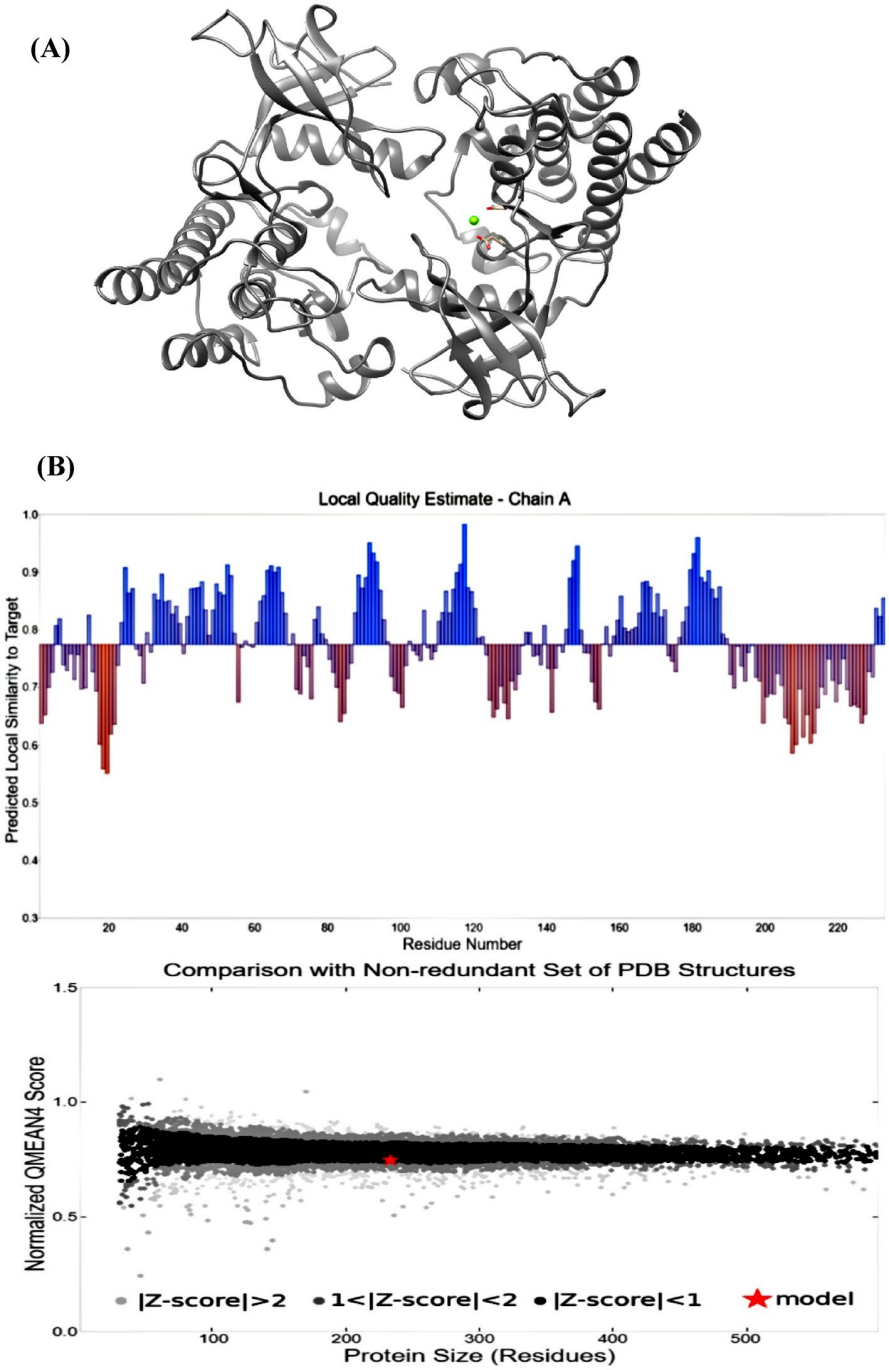
Shows 3D modeling of vaccine construct and validation (**A**) Naïve structure. (**B**) QMEAN server evaluates the structural superiority of cell surface-binding protein.

**Figure 7:**
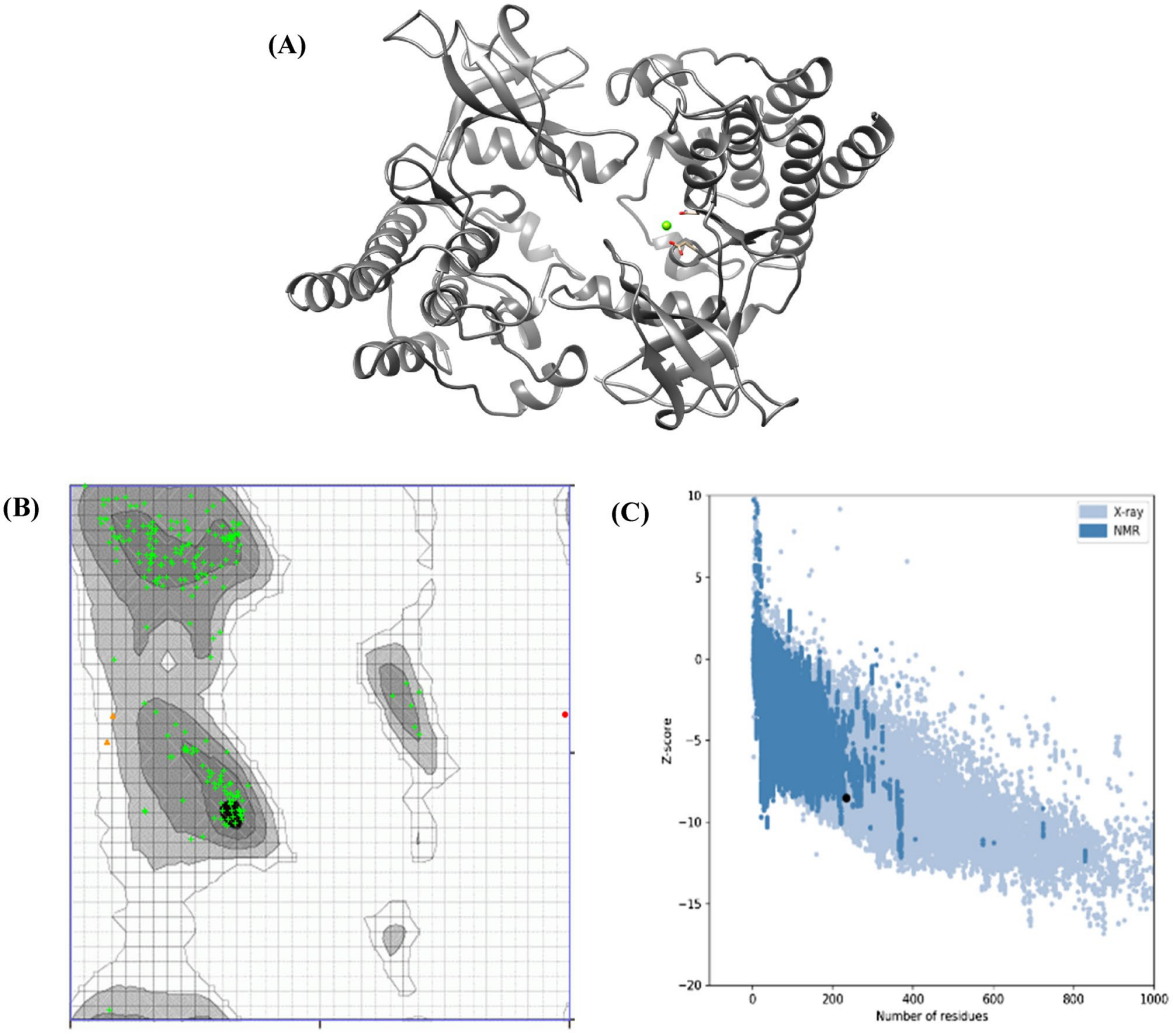
Shows (**A**) GalaxyRefine refined the 3D structure of vaccine construct. (**B**) The refined 3D structure was subjected to a Ramachandran plot analysis showed 98.578% of the residues in favored areas. (**C**) Plot obtained from ProSA-web server.

The obtained 3D structure and the validation analysis are shown in Figs. 6 and 7.

### Physicochemical properties

The obtained results are summarized in Table 5.

**Table 5:** The obtained physicochemical properties.

Characteristics	Finding	Remark
Number of amino acids	275	Suitable
Molecular weight	30.95 kDa	Average
Theoretical pI	9.82	Basic
Chemical formula	C_1422_H_2134_N_386_O_383_S_5_	–
Extinction coefficient (at 280 nm in H_2_O)	58,790 M^−1^ cm^−1^	–
Estimated half-life (mammalian reticulocytes, in vitro)	30 h	–
Estimated half-life (yeast-cells, in vivo)	> 20 h	–
Estimated half-life (*E. coli*, in vivo)	> 10 h	–
Instability index of vaccine	39.84	Stable
Aliphatic index of vaccine	65.75	Thermostable
Grand average of hydropathicity (GRAVY)	− 0.467	Hydrophilic

### Molecular docking study

**Figure 8:**
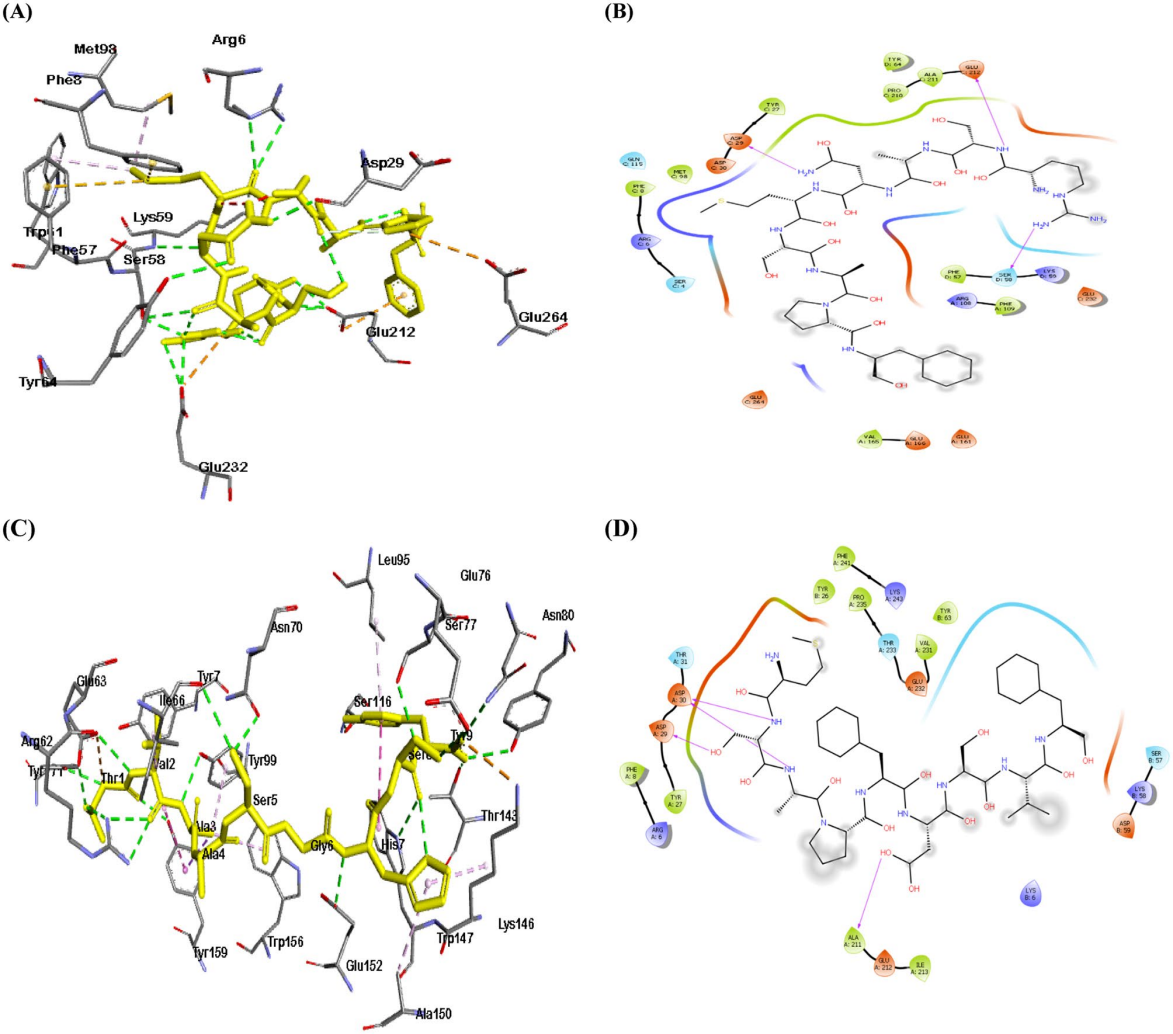
Representive view for Docking analysis of the predicted epitopes: (**A**) 3D view for RSANMSAPF with HLA-A*02:01, (**B**) 2D view for RSANMSAPF with HLA-A*02:01, (**C**) 3D view for MSAPFDSVF with HLA-B*15:01, (**D**) 2D view for MSAPFDSVF with HLA-B*15:01.

**Figure 9:**
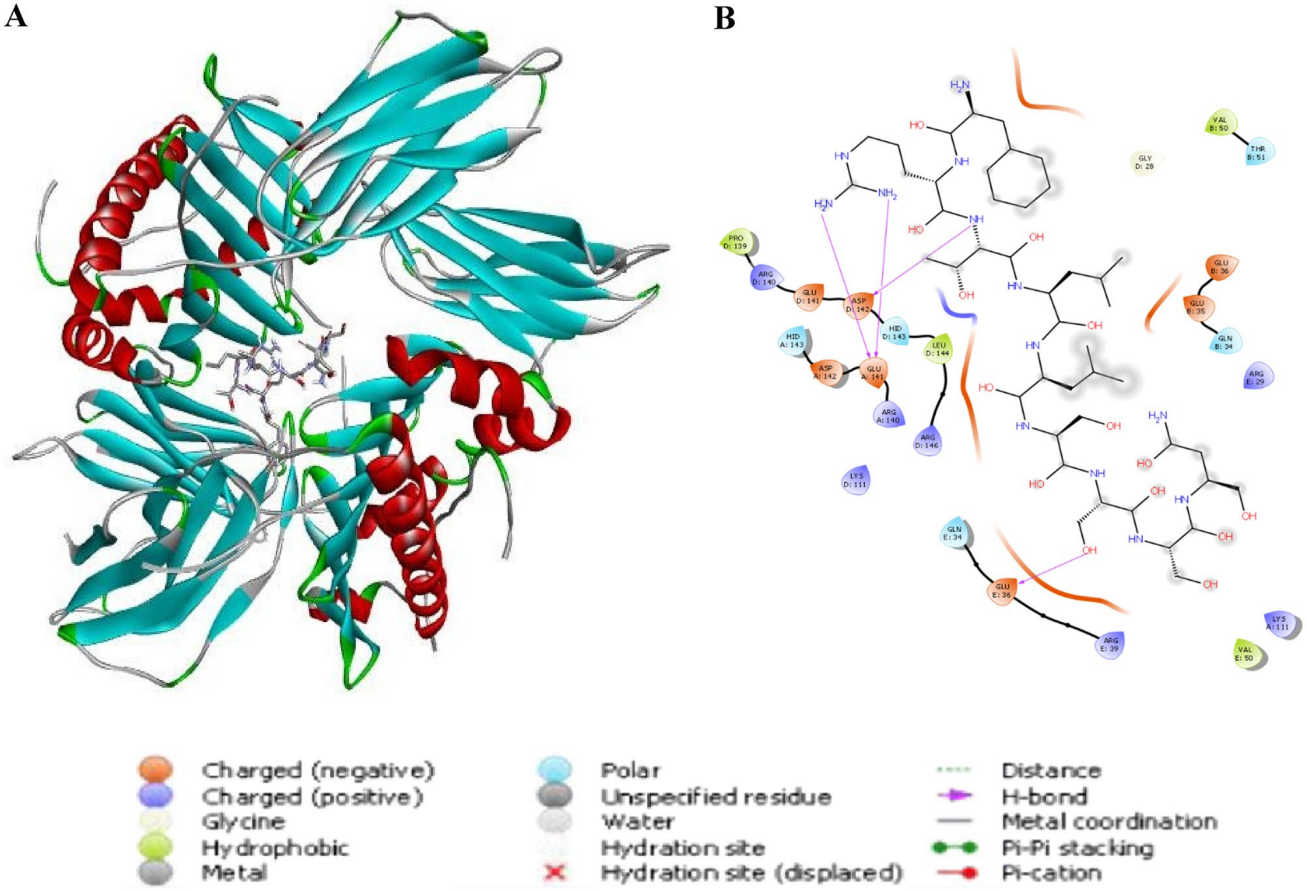
Representive view for Docking analysis of the predicted epitope YVLSTIHIY with HLA-DRB1 (**A**) 3D view, (**B**) 2D view.

The molecular docking was conducted using Glide XP module for the most promising epitopes based on their predicted binding to alleles, IC50, conservation and allergenicity profile (Figs. 8, 9).

### Immune simulation

**Figure 10:**
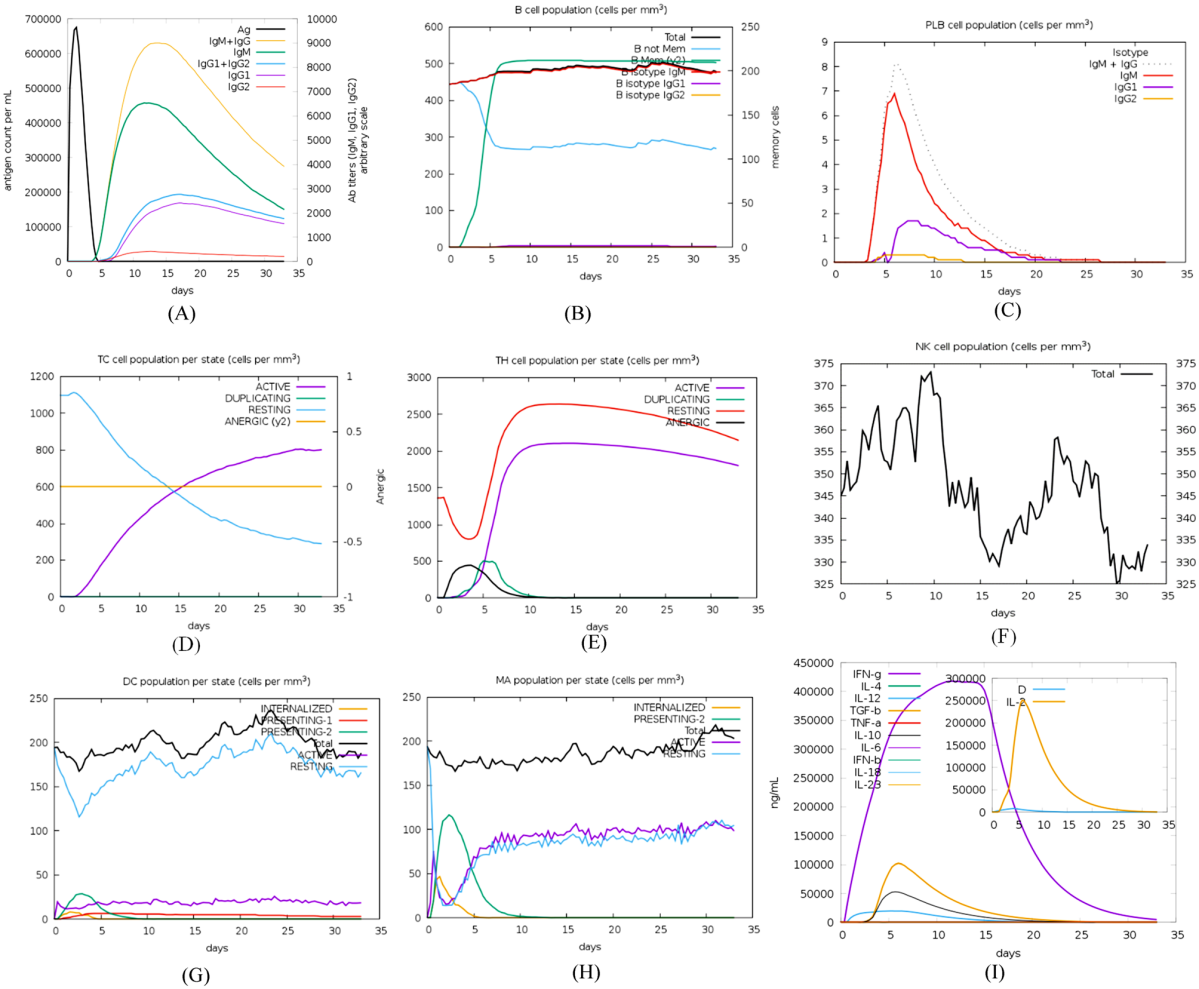
C-ImmSim presentation of an in silico immune simulation with the proposed vaccine. (**A**) immunoglobulin and immunocomplex response to the antigen. (**B**) B lymphocytes: total count, memory cells, and sub-divided in isotypes (IgM, IgG1 and IgG2). (**C**) Plasma B lymphocytes count sub-divided per isotype (IgM, IgG1 and IgG2). (**D**) CD8 T-cytotoxic lymphocytes count per entity-state. (**E**) CD4 T-helper lymphocytes count sub-divided per entity-state (i.e., active, resting, anergic and duplicating). (**F**) Natural Killer cells (total count). (**G**) Dendritic cells. DC can present antigenic peptides on both MHC class-I and class-II molecules. The curves show the total number broken down to active, resting, internalized and presenting the ag. (**H**) Macrophages. Total count, internalized, presenting on MHC class-II, active and resting macrophages. (**I**) The plot displays cytokine levels after injections. The insert plot illustrates the IL-2 level with the Simpson index, D represented by the dotted line. D is a diversity metric. Increase in D over time implies formation of various epitope-specific dominant clones of T-cells. The lower the D value, the lower the diversity.

The obtained results are summarized in Fig. 10.

### Molecular dynamic simulation

**Figure 11:**
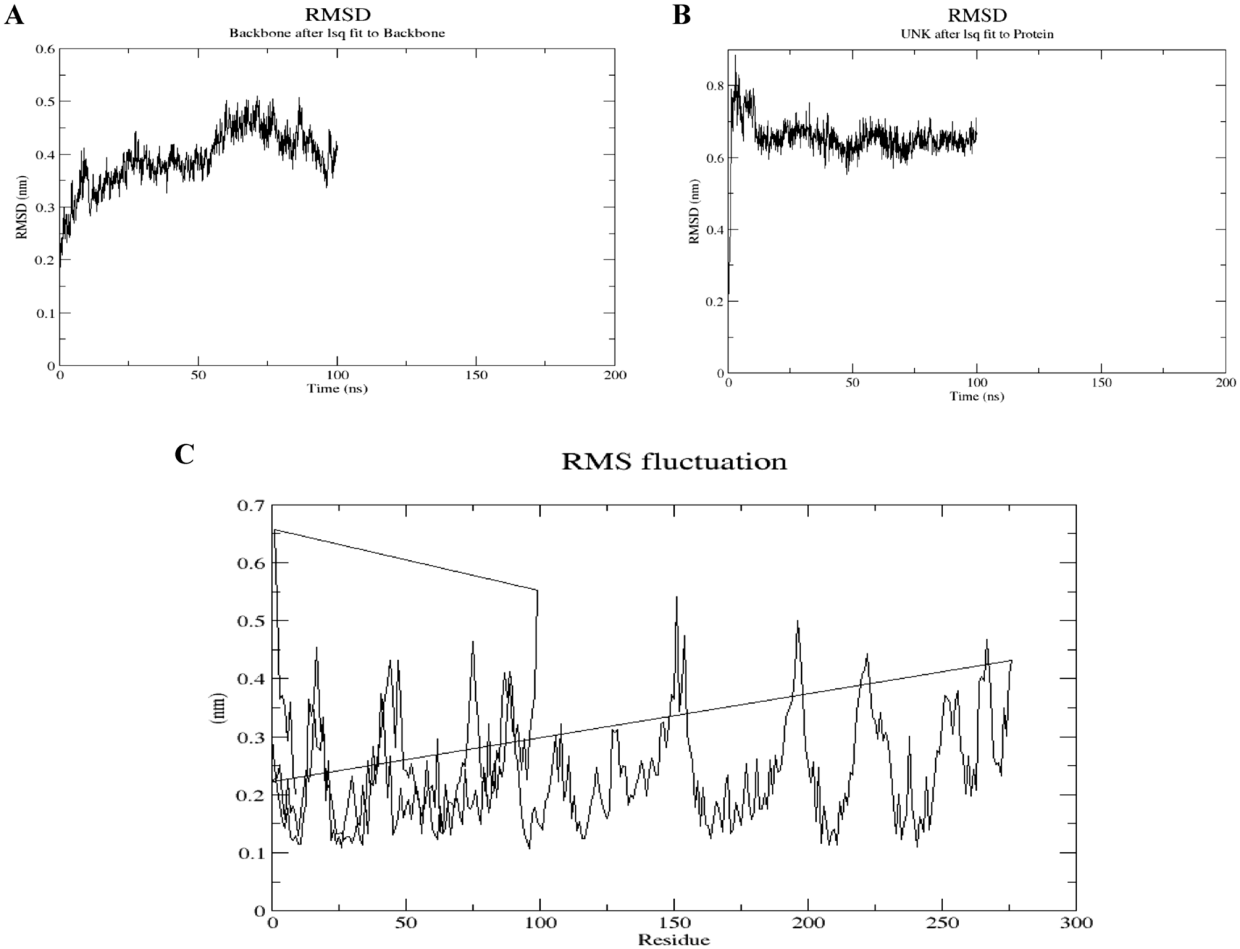
Molecular dynamic simulation, (**A**) The RMSD profiles of the protein backbone, (**B**) The RMSD profiles of the complex, (**C**) The RMSF profile of the complex.

The MD results for the stability and flexibility of the complex are shown in Fig. 11.

### Solubility prediction

**Figure 12:**
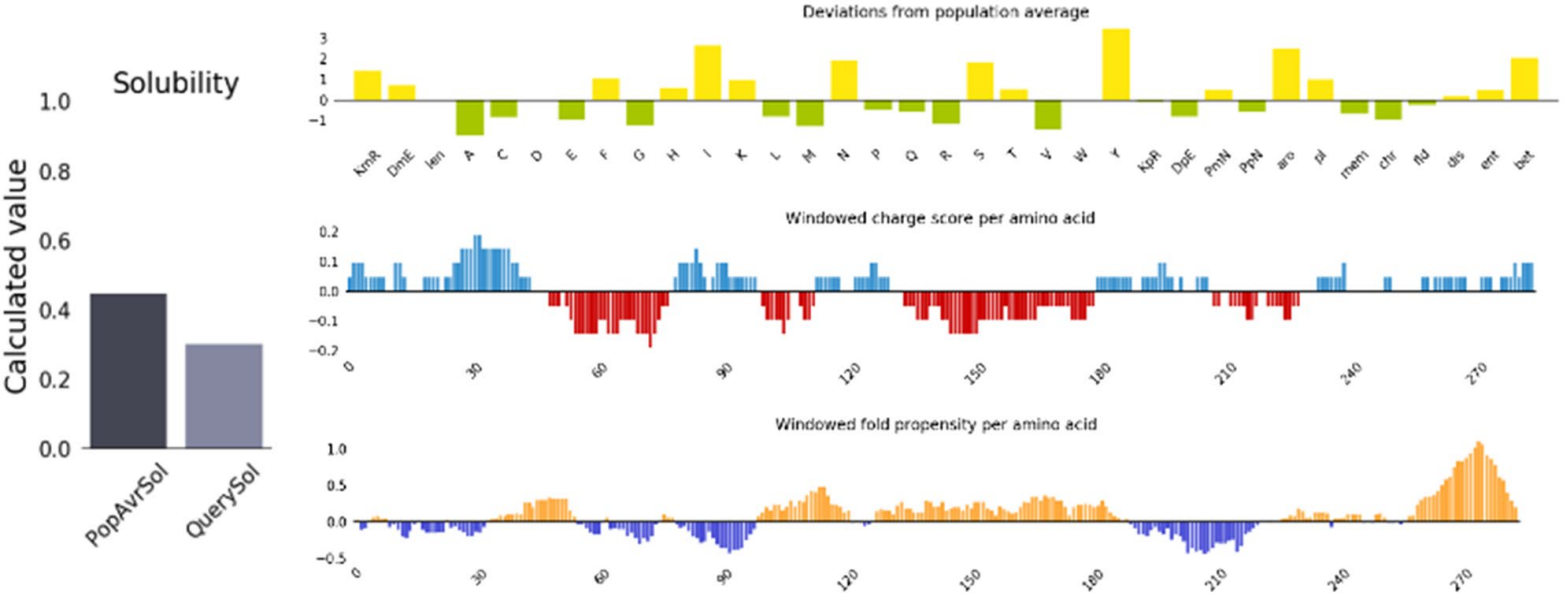
Solubility prediction of designed vaccine by protein-SOL server.

The obtained results are shown in Fig. 12.

## Discussion

The use of immunoinformatics approaches in the construction of potent vaccines against various microorganisms especially viruses, are becoming increasingly acceptable as the first line of vaccine development. In recent years, immunoinformatics-guided approaches have been employed in the design of epitope-based subunits for various diseases^45–47^.

Although it is physiologically less intense than smallpox, monkeypox is a viral zoonotic disease with characteristics similar to those observed in smallpox cases in the past. It is caused by the monkeypox virus possessing hazardous concerns to the public health particularly in Africa^48^. However, it has recently expanded to other parts of the world^49–52^, so it may soon become a global issue. Cell surface-binding protein is responsible of vital biological processes for MPXV such as viral entry into host cell, virion attachment to host cell and Host-virus interaction^53^. Thus, the present study aimed to design a multi-epitope subunit vaccine that can elicit an immune response against MPXV using cell surface-binding protein as a target.

The scientific basis of peptide vaccines is produced by chemical process of synthesizing immunogenic B-cell and T-cell epitopes that can trigger specific antibodies. To create a target molecule immunogenic, a B-cell epitope can be coupled with a T-cell epitope^54,55^.

B-lymphocytes are an important part of the humoral immune system because they produce a wide spectrum of pathogen-specific antibodies that can help in neutralization of the antigens and eliminate viral loads. The BCPRED service was used to assess the antigenic proteome in order to add epitopes into the vaccine design that can potentially activate the B-cells. The server has predicted five conserved epitopes with high scores (Table 2).

The adaptive immune system is triggered by MHC class I epitopes activating Cytotoxic T-cells. The CTL epitopes are further in charge of building long-lasting immunity capable of eradicating circulating virus and infected cells^56^. As a result, MHC class I epitope prediction is critical for vaccine development. In a nutshell, multiple MHC I and II epitopes prediction web-based platforms were used to test the specified viral components of Monkeypox virus. Many epitopes have been reported to interact with MHCI and MHCII alleles from the cell surface- binding protein. Before being nominated for downstream investigations, T cell epitopes were rated using strict in-house criteria. Strong IEDB score, high conservancy, good binding affinity, 9mer for MHC I, 15mer for MHC II, considerably antigenic/immunogenic, non-allergic, non-toxic and topographically accessible to membrane-bound or free antibody are only a few of the selection criteria. Additionally, lower percentile rankings and IC50 values were evaluated for high immunogenicity. According to these guidelines, only two epitopes (RSANMSAPF and MSAPFDSVF) bound to MHC I alleles (Table 3), and one peptide bound to MHC II (YVLSTIHIY) (Table 4) were identified as potential vaccination candidates and selected for further analysis.

The provided epitopes must interact with the majority of ethnic polymorphism MHC I and MHC II alleles with high population coverage scores in order to be regarded as a universal vaccination. The population coverage of the predicted epitopes reacting with T lymphocytes was evaluated in this regard. The suggested epitopes were predicted to have stronger interactions with MHC I and MHC II alleles and were connected to many sets of alleles with high population coverage scores (Fig. 3). This discovery showed that the suggested epitopes might efficiently interact with common human alleles worldwide and cover a sizable population.

Moreover, a peptide must have a range of physiochemical properties in order to operate as an effective vaccine. One of these physiochemical characteristics is molecular weight. Although lymph node exposure is directly proportional, the peptide's half-life inside the body is inversely linked to its molecular weight^57,58^. Peptides larger than 50 kDa are advised for maximum lymph node exposure and half-life in the development of an active immune response. Furthermore, the hydrophobicity and hydrophilicity of the vaccine design have a substantial influence on its efficacy. The ProtParam tool, which is available on the Expasy service, was used to assess the physiochemical attributes. The molecular weight was predicted to be 35.28 kDa with a predicted half-life of 30 h in mammalian reticulocytes.

The vaccine structure's isoelectric point was found to be 9.82, demonstrating the desired vaccine structure's playful nature. According to the ProtParam tool, the structure's instability index was 39.84, indicating that it is a stable protein. The alpha index, which reflects the protein's stability across a large temperature range, was reported to be 65.75 for this developed vaccine construct because the range of this index for stable proteins is less than 40 results. Its GRAVY value is − 0.47, which is a negative value of this index, indicating the nature of the hydrophilic structure of the vaccine, and therefore can interact strongly with water molecules.

According to the secondary structure analysis, the vaccine construct was found to have alpha helices, extended strands, beta turns, and random coiled structures. The refined software greatly improved the three-dimensional structures of the vaccine construct, which illustrated desirable properties on Ramachandran plot prediction model. Still, one of the most difficult problems in structural biology is the detection of errors in experimental and theoretical models of protein structures^59^. As a result, the ProSA program was used to predict the possible structural and modeling errors in the vaccine. ProSA measured the overall quality score for a particular input structure. It plotted the amino acids scores to check the local model quality, and negative values indicate that no parts of the structural model are incorrect. The outcome has been shown in a plot that included the scores of all experimentally protein chains publicly available in the Protein Data Bank^59^. This study's projected vaccine design got a Z-score of -8.5, suggesting the satisfaction of the model as a vaccination against MPXV (Figs. 5, 6, 7).

The interactions between antigenic molecules and immune receptor molecules are critical for efficient antigenic transport and immune response activation^60^. In order to study the probable interactions, binding energy, and poses, docking analysis was performed between immune receptor molecules and the developed epitopes. Docking analysis was performed utilizing Schrödinger which evaluates postures, including their solid molecular surface display (Figs. 8, 9).

The creation of H-bonds in the protein–ligand complex is a crucial metric for determining the stability of the conformation across the simulation period^61^. The 2D view was utilized to investigate the interactions and bonding with MHC molecules (Fig. 8). RSANMSAPF and MSAPFDSVF were bonded to the groove of HLA-A*02:01 and HLA-B*15:01 with binding energies of − 8.6 kcal/mole and − 7.5 kcal/mole, respectively. RSANMSAPF established three hydrogen bonds with ASP29; SER58; and GLU212 residues, while MSAPFDSVF established four hydrogen bonds with ASP29, ASP30, and ALA211 residues. While YVLSTIHIY was bound to the groove of HLA-DRB1 with binding energy of − 7.1 kcal/mole (Fig. 9).

The obtained immunological simulation were found to be consistent with the typical immune responses^62,63^; as there was a general rise in the produced immune responses after repeated antigen exposure. Memory B-cells and T-cells were clearly developing. An intriguing finding was that IFN- and IL-2 levels increased after the initial injection and stayed at high levels after repeated antigen doses. This implies a high number of TH cells and, as a result, efficient Ig generation, which supports a humoral response (Fig. 10).

Using WebGro, the complex gave the best binging energy was 100 ns simulated to study the dynamic atomic interactions of the vaccine inside the active site. To examine the configurational alterations that take place when a ligand is driven to fit, MD modeling provides a very precise method. The protein/complex system is computationally developed with WebGro for a brief 100 ns timespan using classical mechanics, and the configurational stability or binding affinity of a ligand is evaluated during the simulation trajectory. Here, as shown in Fig. 11, the RMSD plot of the complex initially showed very low variations till 20 ns ranging 0.6 to 0.8 nm followed by stable conformation till 100 ns. This stability could be attributed to the higher number of stable bonding between compound and the target protein.

Afterwards, the RMSF (root mean square fluctuation) value was calculated to investigate the structural flexibility of protein’s backbone atoms (Fig. 11). Obtained results indicated no high fluctuation and the flexibility of the complex (RMSF ≤ 0.5 nm). This result implies that the designed vaccines can strongly interact with the immune receptors.

The protein's solubility is another important factor for various functional and biochemical studies when overexpressed in the *E. coli* host^64^. As highly soluble proteins during downstream processing exhibit ease of purification, this proves better post-production outcomes^65^. The predicted vaccine was revealed to be soluble when over-expressing in *E. coli* (Fig. 12).

As a conclusion of these promising results, constructing a vaccine based on the proposed peptides seems to be a high priority, with the potential to be extensively deployed as a universal epitope-based peptide vaccine against MPXV.

## Conclusion

The current work was planned and then manufactured a multi-epitope vaccine that can evoke an immunological response against MPXV utilizing cell surface-binding protein as a target in order to develop a novel vaccine that is both safe, effective, and almost free of side effects. The most promising peptides in the cell surface protein were then selected applying a rigorous procedure and used for vaccine design. As a result, our study will aid in the development of appropriate therapeutics and prompt the development of future vaccines against MPXV, which could serve as an important milestone in the production of an antiviral vaccine against MPXV.

## Data Availability

The datasets generated and/or analysed during the current study are available within the manuscript and in UniProt (https://www.uniprot.org/) with accession number Q8V4Y0.
